# Acylcarnitines in Cancer Metabolism: Mechanistic Insights and Stratification Potential

**DOI:** 10.3390/cancers18040713

**Published:** 2026-02-23

**Authors:** Hwa Pyoung Lee, Jieun Oh, Nury Lee, Yujin Jung, Jisu Yum, Minsu Kim, Maro Yoo, Jae Gwang Park, Jae Youl Cho

**Affiliations:** 1 InnoBation Bio, 189 Seongam-ro, Seoul 03929, Republic of Korea; hplee@innobationbio.com (H.P.L.); nr@innobationbio.com (N.L.); jyj@innobationbio.com (Y.J.); jisu@innobationbio.com (J.Y.); 2 Department of Integrative Biotechnology, Sungkyunkwan University, Suwon 16419, Republic of Korea; martia96@skku.edu (J.O.); tantang123@g.skku.edu (M.K.); 3 Center for Liver and Pancreatobiliary Cancer, Hospital, National Cancer Center, Goyang 10408, Republic of Korea; yoomaro@ncc.re.kr

**Keywords:** acylcarnitine, cancer metabolism, fatty acid oxidation, metabolic reprogramming, non-invasive biomarkers, therapeutic targets

## Abstract

Metabolic reprogramming constitutes a fundamental hallmark of malignancy, enabling cancer cells to sustain proliferation and survival under physiological stress. While aerobic glycolysis is well characterized, fatty acid oxidation (FAO) has emerged as a decisive driver of oncogenic progression and therapeutic resistance. Acylcarnitines (ACs), obligatory intermediates for the mitochondrial transport of long-chain fatty acids, have transcended their traditional categorization as passive metabolic byproducts to function as potent signaling entities and functional readouts of mitochondrial oxidative throughput. This review delineates the AC metabolic axis in oncology, examining the coordinated biochemical machinery, including the carnitine palmitoyltransferase (CPT) system, carnitine–acylcarnitine translocase (CACT; SLC25A20), and organic cation/carnitine transporter 2 (OCTN2), that governs cellular AC homeostasis. We further evaluate the clinical utility of altered AC profiles as non-invasive biomarkers for early diagnosis and risk stratification across diverse malignancies, highlighting their capacity to reflect metabolic bottlenecks and flux dynamics. Additionally, we scrutinize therapeutic strategies targeting the AC-FAO axis, demonstrating how the inhibition of key transporters and enzymes sensitizes tumors to conventional chemotherapy and immunotherapy. Ultimately, deciphering the systemic and spatial dynamics of ACs remains essential for advancing precision metabolic oncology and developing personalized therapeutic strategies based on metabolic profiling.

## Simple Summary

Cancer cells rewire their metabolism to fuel rapid growth and survival. Recently, their dependence on fatty acids as a critical energy source has been recognized as a key survival strategy. This review explores the role of “acylcarnitines”, which are essential molecules acting as shuttles to transport fatty acids into mitochondria for energy production. We explain how cancer cells utilize this transport system to withstand metabolic stress and how specific acylcarnitines leak into the bloodstream. These leaked molecules can serve as non-invasive biomarkers, allowing for cancer detection through blood profiling. Furthermore, we discuss how blocking this transport system can starve cancer cells and overcome resistance to chemotherapy. This overview highlights acylcarnitines as promising targets for both early diagnosis and novel metabolic therapies.

## 1. Introduction

Cancer cells reconfigure fundamental biological paradigms by evading immune checkpoints and reshaping their microenvironment to gain a survival advantage [1,2,3]. Tumor tissues orchestrate the extensive rewiring of core metabolic networks, including fatty acid oxidation (FAO) and glucose utilization, to monopolize energetic resources and sustain unconstrained proliferation [2,4]. Since the initial linkage of metabolic derangements to malignant growth, systematic dissection of both canonical and non-canonical pathways has elucidated the metabolic requirements for cell proliferation [4,5]. Consequently, characterizing these cancer-specific metabolic traits has emerged as a pivotal strategy for identifying novel therapeutic vulnerabilities.

Concomitant advances in analytical chemistry and metabolomics have enabled the high-resolution profiling of small molecules, facilitating the identification of specific metabolites as candidate biomarkers for cancer detection and monitoring. Among these, acylcarnitines (ACs) have garnered particular interest as non-invasive indicators of tumor progression, patient prognosis, and therapeutic response [6,7]. Accordingly, metabolite-based profiling offers critical insights into the biochemical state of tumors, facilitating more precise diagnostic and prognostic evaluations.

ACs constitute a class of fatty acid derivatives comprising diverse acyl chains esterified to the hydroxyl group of L-carnitine, with their physiological relevance documented since the 1960s [8]. These metabolites play an obligatory role in mitochondrial fatty acid β-oxidation, specifically by shuttling long-chain fatty acids into mitochondria, where they are converted into substrates for cellular energy production [9,10]. Distinct long-chain ACs are associated with specific pathological states, and select AC species are routinely utilized as biomarkers for inborn errors of metabolism and other metabolic disorders [6,10]. More recently, accumulating evidence has elucidated the links between AC profiles and cancer biology, highlighting their potential involvement in tumor initiation and progression [11].

Driven by advances in analytical platforms and data science, non-invasive diagnostic strategies based on biofluids are rapidly evolving through the integration of metabolomics, lipidomics, and bioinformatics [12,13]. Lipids serve as attractive analytes because they closely mirror disease stage and dynamic metabolic remodeling, thereby fueling interest in lipidomics-based approaches for cancer detection [14,15]. In this context, current investigations focus on how AC kinetics and profile alterations contribute to molecular stratification and risk assessment of specific diseases, while applying advanced computational models to rigorously assess their accuracy [16,17].

This review focuses on the role of ACs as central energy-related metabolites in cancer metabolism and delineates their functional contributions to tumorigenesis and cancer progression. A deeper understanding of AC metabolic alterations is expected to underpin the design of comprehensive clinical strategies for non-invasive cancer diagnosis and longitudinal disease monitoring in patients with malignancies.

## 2. Biochemical Pathways of ACs

### 2.1. Synthesis and Diversity of ACs

ACs originate from the covalent esterification of acyl groups to the hydroxyl moiety of L-carnitine, functioning as obligatory intermediates in cellular metabolism. These species encompass a diverse spectrum of acyl chains, ranging from short- to long-chain variants, alongside isomers derived from branched-chain fatty acid and amino acid catabolism. Synthesis proceeds primarily via the conjugation of acyl-CoA with carnitine. This reaction facilitates the translocation of acyl groups across mitochondrial membranes, a prerequisite for β-oxidation and the subsequent regeneration of acetyl-CoA and associated metabolic derivatives [18]. Within this framework, AC pools operate as a critical buffering and trafficking system, particularly when the acyl-CoA supply transiently exceeds downstream oxidative capacity.

### 2.2. Enzymes and Transporters

A coordinated network of enzymes and transporters orchestrates AC metabolism. Carnitine palmitoyltransferase 1 (CPT1), localized to the outer mitochondrial membrane, catalyzes the conversion of cytosolic long-chain acyl-CoA to AC, thereby enabling entry into the mitochondrial intermembrane space. CPT1 constitutes a primary rate-limiting checkpoint for long-chain fatty acid influx and is canonically allosterically inhibited by malonyl-CoA, thereby coupling FAO capacity to systemic nutrient signaling. Carnitine–acylcarnitine translocase (CACT; SLC25A20) subsequently mediates the translocation of ACs across the inner mitochondrial membrane. Following import, carnitine palmitoyltransferase 2 (CPT2) within the matrix reconverts the acyl group to acyl-CoA to initiate β-oxidation [19,20]. Concurrently, organic cation/carnitine transporter 2 (OCTN2, SLC22A5) governs carnitine uptake at the plasma membrane, ensuring the maintenance of systemic and cellular carnitine homeostasis [21,22].

### 2.3. AC-Mediated Fatty Acid β-Oxidation

To fuel mitochondrial β-oxidation, ACs facilitate the obligatory shuttling of long-chain fatty acids into the matrix. These fatty acids, initially activated as acyl-CoA, undergo conversion to ACs for translocation across the inner membrane, followed by reconversion to acyl-CoA within the matrix to drive sequential β-oxidative cycles. These cycles cleave two-carbon units to yield acetyl-CoA, nicotinamide adenine dinucleotide (NADH), and flavin adenine dinucleotide (FADH_2_) to satisfy cellular energy demands [23,24]. Because the reducing equivalents derived from β-oxidation require processing by the electron transport chain, constraints on mitochondrial respiration can precipitate a metabolic imbalance favoring AC accumulation. Accordingly, AC profiles frequently function as a metabolic readout reflecting the stoichiometry between fatty acid influx and oxidative throughput.

## 3. Role of AC in Cancer Metabolism

The AC axis is a central metabolic nexus linking fatty acid availability to mitochondrial bioenergetic capacity. Beyond serving as transport intermediates, AC species provide a functional readout of the quantitative balance between lipid influx and mitochondrial oxidative throughput. When fatty acid influx exceeds the catalytic capacity of β-oxidation and/or the tricarboxylic acid (TCA) cycle, the accumulation of specific AC species more often indicates mitochondrial bottlenecks or respiratory constraints than increased oxidative flux. Therefore, AC dynamics can illuminate tumor metabolic plasticity and dependence on lipid oxidation, but they require context-aware interpretation.

Most current evidence is derived from steady-state metabolomics, which cannot reliably disentangle real-time FAO flux from dynamic mitochondrial processes. In this setting, AC accumulation frequently reflects metabolic congestion rather than pathway activation, warranting caution when inferring enhanced FAO activity [25,26]. Where feasible, these inferences are best strengthened by complementary flux-focused measurements, such as stable isotope tracing-based FAO flux analysis and/or mitochondrial respiration readouts [27]. Building on this interpretive framework, the following sections summarize cancer-context features of fatty acid β-oxidation and integrate AC dynamics with energy metabolism, redox homeostasis, therapeutic resistance, and regulated cell death.

### 3.1. Distinct Features of Fatty Acid β-Oxidation and AC Dynamics in Malignant Cells

In contrast to the relatively homeostatic profile of quiescent tissues, malignant cells can display marked metabolic plasticity in fatty acid β-oxidation (FAO). Although the Warburg effect has historically dominated the field, accumulating evidence indicates that, in specific tumor contexts, including subsets of ovarian and prostate cancers, FAO can serve as a major bioenergetic and stress-adaptive program supporting survival and metastatic progression [28,29].

A characteristic feature of cancer-associated FAO is a stoichiometric mismatch between fatty acid uptake and mitochondrial oxidative throughput. When fatty acid influx exceeds mitochondrial oxidative capacity, long-chain AC species accumulate, reflecting incomplete oxidation and or respiratory constraint rather than efficient bioenergetic turnover [9,25]. Emerging work further suggests that metabolic reprogramming, characterized by aberrant lipid accumulation and oxidation, acts as a critical driver that modulates pro-tumorigenic pathways and contributes to remodeling of the tumor microenvironment (TME) [30].

Consistent with this rewiring, many malignant cells exhibit increased CPT1A activity and or expression to sustain mitochondrial fatty acid import and AC kinetics, particularly under metabolic stress or hypoxia where glucose oxidation becomes constrained [29]. Collectively, context-dependent engagement of the AC–FAO axis may help malignant cells maintain energy balance and redox fitness within nutrient- and oxygen-limited tumor niches [31].

### 3.2. Role in Energy Metabolism

ACs execute central functions within the bioenergetic framework of cancer cells. Building on the foundational investigations by Konrad Bloch and Feodor Lynen, and the seminal concept of the Warburg effect, contemporary studies have rigorously dissected metabolic reprogramming in oncology [11,32]. These metabolites are generated in peroxisomes and mitochondria under the regulation of the coordinated CPT1/2 and CACT machinery [33,34]. The acyl moiety dissociates from AC to recombine with CoA, thereby serving as the primary substrate for mitochondrial fatty acid β-oxidation. The resultant acetyl-CoA subsequently enters the TCA cycle to drive adenosine triphosphate (ATP) generation [24,34].

**Figure 1:**
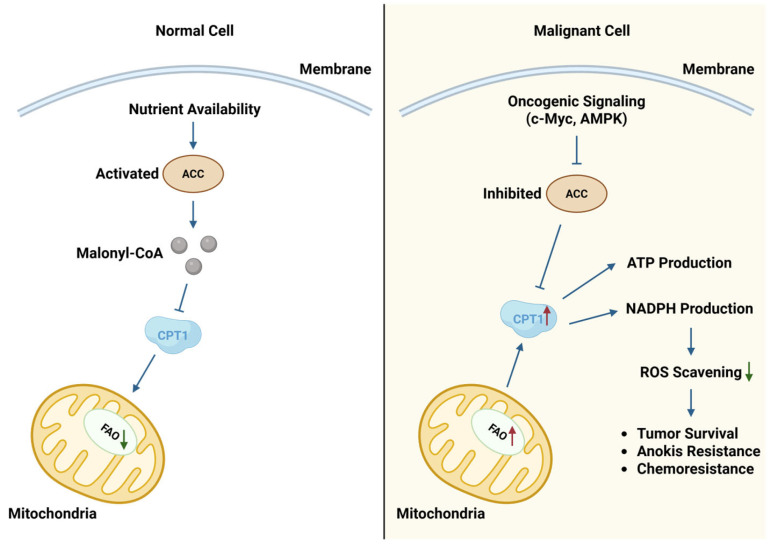
Differential regulation and functional outcomes of FAO in normal versus malignant cells. In physiological quiescence (**Left panel**), FAO is governed by stringent feedback loops mandated by nutrient availability. Mechanistically, ACC biosynthesizes malonyl-CoA, which imposes allosteric inhibition on CPT1, thereby obviating futile cycling between lipid synthesis and oxidation. In sharp contrast, within malignant cells (**Right panel**), this regulatory circuit is frequently uncoupled. Orchestrated by oncogenic signaling (e.g., c-Myc, AMPK), cancer cells constitutively overexpress CPT1 while abrogating ACC activity, permitting sustained FAO flux even under nutrient-rich conditions. This metabolic reprogramming endows cells with a dual survival advantage: fueling bioenergetics (ATP) while concurrently supplying NADPH for redox buffering, thus attenuating oxidative stress to sustain anoikis resistance during metastasis and chemoresistance. [Created in BioRender. Hwa Pyoung, L. (2026) https://BioRender.com/gd617rb].

ACs are stratified by acyl chain length into short-, medium-, and long-chain forms. Short-chain variants typically derive from glucose, amino acid, and fatty acid catabolism, whereas medium- and long-chain species emerge predominantly in association with mitochondrial fatty acid handling and β-oxidation [35,36]. The import of long-chain ACs into the mitochondrial matrix necessitates precise coordination among CPT1/2 and CACT, constituting a pathway integral to cancer cell metabolic plasticity and energy provisioning [11,33]. In this context, AC profiles can reflect the balance between fatty acid influx and mitochondrial oxidative capacity, which is frequently reshaped in malignant cells, and should be interpreted accordingly [37]. Ultimately, AC metabolism underpins the capacity of cancer cells to rewire pathways for energy and biomolecule synthesis, thereby reinforcing tumor growth and survival. Consequently, the metabolic governance of FAO displays a stark divergence between physiological homeostasis and the malignant state, as delineated in Figure 1.

### 3.3. Redox Balance and Reactive Oxygen Species (ROS) Regulation

ROS function as critical signaling molecules for redox balance, arising continuously from mitochondrial electron transport, peroxisomal β-oxidation, and endoplasmic reticulum protein oxidation [38,39]. While physiological ROS levels modulate signaling cascades, excess accumulation precipitates cellular damage and apoptosis [40,41,42]. Cancer cells, characterized by accelerated proliferation and elevated fatty acid turnover, exhibit heightened ROS production that influences oxidative injury, signaling networks, and metabolic rewiring [39,43].

CACT, embedded in the inner mitochondrial membrane, shuttles acyl groups to support mitochondrial fatty acid import and FAO-linked ATP production [37,44]. However, the interplay between AC dynamics and ROS generation is intrinsically reciprocal. Excess accumulation of long-chain AC species (e.g., C16) can be lipotoxic; through their amphiphilic properties, these metabolites compromise mitochondrial membrane integrity, triggering membrane depolarization and propagating secondary ROS release [45]. This membrane perturbation may further destabilize electron transport, amplifying ROS generation [46]. As a counter-regulatory mechanism against this oxidative stress, cysteine residues on CACT undergo glutathionylation in response to elevated ROS flux. This redox-sensitive post-translational modification reduces CACT transport activity, limiting additional fatty acid influx and thereby restoring redox homeostasis and mitigating a deleterious feed-forward cycle of ROS accumulation [44].

Taken together, AC species function as active modulators of the cellular redox state rather than inert metabolic substrates. Malignant cells maintain stringent control over AC homeostasis to equilibrate bioenergetic requirements with the potential for ROS-induced cytotoxicity, a balance that can support survival and may contribute to therapeutic resistance.

### 3.4. Contribution to Therapy Resistance

FAO has emerged as a compelling therapeutic target for overcoming drug resistance across diverse malignancies. This pathway modulates the efficacy of various modalities, ranging from conventional chemotherapeutics to targeted agents and immunotherapies [34,47]. In malignant cells, heightened fatty acid synthesis and accumulation frequently drive mitochondrial FAO hyperactivity, particularly under microenvironmental or therapeutic stress. Notably, this metabolic shift depends on the biosynthesis and intracellular trafficking of ACs. As obligatory intermediates, ACs facilitate the shuttle of long-chain fatty acids into the mitochondrial matrix via the rate-limiting enzyme CPT1, thereby sustaining oxidative phosphorylation and supporting mitochondrial energy and redox demands [11,48].

Recent studies have reported alterations in cytoplasmic AC pools in cancer cells, particularly under conditions of metabolic or therapeutic stress [35,49]. Accumulation of specific AC species has been observed in association with enhanced FAO activity or with imbalances between fatty acid influx and mitochondrial oxidative capacity [10,23]. These metabolic changes are frequently described in tumors exposed to microenvironmental constraints, such as hypoxia or nutrient limitation, as well as during exposure to anticancer therapies [50].

Functionally, engagement of the AC–FAO axis can promote drug tolerance by maintaining ATP supply, buffering therapy-induced oxidative stress, and limiting lipotoxic pressure from excess acyl groups [51,52]. Such adaptations may reduce apoptosis-inducing stress signals during treatment and support the persistence of drug-tolerant cell states [53].

### 3.5. ACs in Regulation of Cell Death: Apoptosis and Ferroptosis

Transcending their primary bioenergetic roles, ACs and their cognate metabolic enzymes function as important determinants of cell fate by modulating apoptotic and ferroptotic signaling axes. Excessive accumulation of long-chain AC species can induce potent lipotoxicity, compromising mitochondrial membrane integrity and precipitating intrinsic apoptosis. For instance, under stringent metabolic conditions such as tumor hypoxia, constraints within the FAO machinery can promote the accumulation of incompletely oxidized lipid intermediates, specifically long-chain AC species [11,19].

Conversely, cancer cells often upregulate the CPT and AC axis to limit this lipotoxicity. By efficiently shuttling fatty acids into the mitochondria for oxidation, the CPT system reduces the accrual of deleterious lipid intermediates, thereby dampening apoptotic signaling and sustaining tumor viability under metabolic stress [11]. In parallel, recent investigations have delineated a novel nexus between the FAO-AC axis and ferroptosis, an iron-dependent regulated cell death pathway orchestrated by lipid peroxidation.

CPT1A has emerged as a critical negative regulator of ferroptosis, particularly in certain cancer stem cell contexts. Mechanistically, CPT1A overexpression can reduce the availability of polyunsaturated fatty acids susceptible to peroxidation while concurrently activating the nuclear factor erythroid 2-related factor 2 (NRF2) antioxidant axis, thereby conferring a ferroptosis-resistant phenotype [47]. In addition, pharmacological blockade of CPT1A has been demonstrated to potentiate the efficacy of ferroptosis-inducing agents, suggesting that modulating AC-dependent flux may provide a practical strategy to engage ferroptosis in refractory cancers [54].

## 4. Mechanistic Landscapes and Clinical Utility of AC Profiles Across Cancer Types

**Figure 2:**
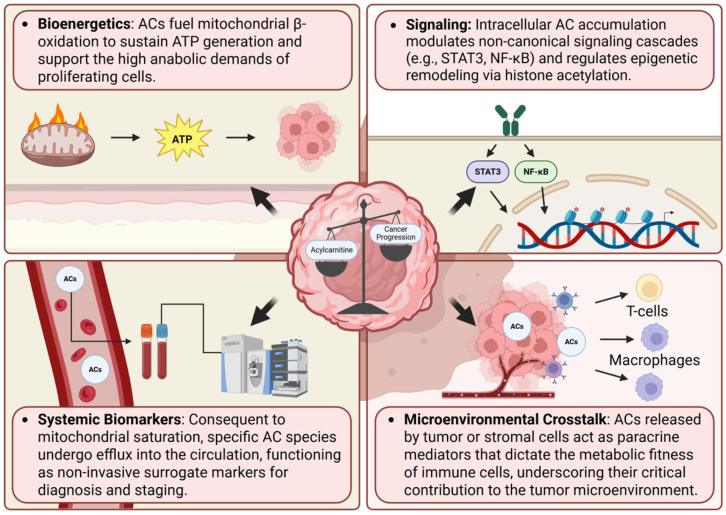
The multifaceted roles of ACs in cancer progression and clinical utility. Transcending their canonical function as mitochondrial fuel shuttles, ACs orchestrate pleiotropic effects on tumorigenesis. (1) Bioenergetics: ACs drive mitochondrial β-oxidation to sustain ATP generation and support the robust anabolic demands of proliferating cells. (2) Signaling: Intracellular AC accrual modulates non-canonical signaling cascades (e.g., STAT3, NF-κB) and coordinates epigenetic remodeling via histone acetylation. (3) Systemic Biomarkers: Consequent to mitochondrial saturation, specific AC species undergo efflux into the circulation, serving as non-invasive surrogate markers for diagnosis and prognostic staging. (4) Microenvironmental Crosstalk: ACs released by tumor or stromal cells function as paracrine mediators that dictate the metabolic fitness of immune cells, underscoring their critical contribution to the TME. [Created in BioRender. Hwa Pyoung, L. (2026) https://BioRender.com/7l39yys].

While the fundamental principles of AC-mediated metabolic rewiring constitute a broad framework, specific AC signatures and their subsequent clinical utility exhibit significant diversity depending on the tumor origin and microenvironment. Building on these principles, this section delineates how AC fluctuations manifest across major malignancies and evaluates their potential as clinically actionable biomarkers for early detection, staging, and prognostic assessment. Beyond these specific applications, the multifaceted contributions of ACs in bioenergetics, signaling cascades, systemic biomarker readouts, and microenvironmental crosstalk are schematically delineated in Figure 2.

### 4.1. Glandular and Reproductive Malignancies: ACs as Mediators of Hormone-Driven Proliferation

In cancers of the reproductive system, ACs function not merely as metabolic byproducts but as active participants in supporting the high energy demands of hormone-driven growth.

#### 4.1.1. Ovarian Cancer

Metabolomic profiling has identified short-chain ACs, specifically C3, as candidate biomarkers for early-stage detection [55]. C2 concentrations are significantly elevated in endometrioid and clear cell subtypes, where they correlate with a higher risk of disease progression [56]. A landmark finding in epithelial ovarian cancer is the dramatic accumulation of C4; primary tumors exhibit a 3.62-fold increase, which escalates to 7.88-fold in metastatic sites [57]. From a clinical perspective, pelvic fluid analysis has demonstrated high diagnostic potential. A 24-metabolite panel, including C16, effectively distinguished patients from controls with an area under the curve (AUC) of 0.992 (sensitivity 97.5%, specificity 97.5%), underscoring the utility of proximate biofluids for precise tumor detection and staging [58].

Across studies, ovarian cancer shows coordinated perturbations in short- and long-chain AC profiles that align with clinical stage, histological subtype, and metastatic progression. These observations support the potential use of AC profiling for early detection, staging, and risk stratification.

#### 4.1.2. Breast Cancer

The circulating AC pool in breast cancer reflects a complex interplay between hormonal status and systemic metabolism. Higher C2 levels are positively associated with breast cancer risk, particularly in postmenopausal women not receiving hormone replacement therapy [59]. In estrogen receptor-positive (ER+) subtypes, independent associations exist for C5 and C6-DC, whereas medium-chain species like C10:1 and C10:2 are associated with a reduced risk, suggesting a protective role through more efficient lipid processing [60,61]. These metabolic shifts translate into tangible clinical utility; a serum panel of three medium-chain ACs (C12, C14, and C14:2) combined with 9,12-linoleic acid differentiated cancer cases from healthy controls with an AUC of 0.839 (sensitivity 83%, specificity 81%). This underscores the potential of non-invasive AC profiling as a mechanistic indicator for breast malignancy [62].

Across cohorts, breast cancer is associated with distinct AC signatures influenced by hormonal status and systemic metabolic homeostasis. These reported patterns suggest that AC profiling may help capture tumor aggressiveness and metabolic heterogeneity in minimally invasive samples.

#### 4.1.3. Prostate Cancer

Fatal cases of prostate cancer are characterized by distinct elevations in C4 concentrations [63]. Mechanistically, the accumulation of C16 induces a pro-inflammatory state and enhances calcium influx, which in turn activates signaling pathways that promote bone metastasis and resistance to androgen deprivation therapy [64,65]. Beyond its mechanistic role, AC profiling offers significant value for clinical stratification. The accumulation of specific ACs, C4 and C16, serves as a metabolic indicator of aggressive phenotypes, providing a window for monitoring disease severity and identifying patients at higher risk of therapeutic resistance [63,64].

Overall, elevations in C4 and C16 are repeatedly associated with aggressive disease phenotypes and resistance to androgen deprivation therapy. This association supports the potential role of AC profiling in stratification and longitudinal monitoring of disease severity.

### 4.2. Digestive and Hepatobiliary Cancers: Biomarkers of Metabolic Bottlenecks and Mitochondrial Overload

Gastrointestinal and liver cancers often exhibit metabolic bottlenecks where the supply of fatty acids exceeds mitochondrial processing capacity, accompanied by increases in specific AC intermediates that reflect incomplete oxidation or respiratory constraint.

#### 4.2.1. Hepatocellular Carcinoma

Hepatocellular carcinoma is defined by a significant metabolic shift where long-chain ACs (C18:1, C18:2) increase while medium-chain species (C8, C10) decline [66]. This pattern frequently arises from the downregulation of CPT2, creating a metabolic bottleneck by preventing the complete conversion of long-chain fatty acids into shorter intermediates. In non-alcoholic fatty liver disease-associated hepatocellular carcinoma, C18:1 directly activates oncogenic signaling, reinforcing its role as both a biomarker and a driver of malignancy [67,68,69,70]. These biochemical signatures offer substantial clinical utility; hepatocellular carcinoma displays etiology-specific metabolic profiles where steatohepatitis-derived cases feature elevated AC levels, while non-steatohepatitis patients exhibit serum AC reductions. Applying a 5.088 μmol/L cutoff for serum ACs yielded an AUC of 0.925 (sensitivity 92.9%, specificity 89.5%), demonstrating superior diagnostic potential compared to traditional markers such as alpha-fetoprotein (AFP) in identifying specific metabolic subtypes [70].

Taken together, this AC pattern, featuring increased long-chain species with reduced medium-chain species, is consistent with impaired FAO and mitochondrial overload, supporting AC profiling for metabolic subtyping and diagnostic evaluation.

#### 4.2.2. Colorectal Cancer

Colorectal cancer patients exhibit enriched levels of C2, C14, and unsaturated species such as C14:1 and C14:2 [71,72,73]. Beyond absolute concentrations, the C4/C3 and C5/C2 ratios serve as robust diagnostic signatures, reflecting a profound imbalance in mitochondrial flux that correlates with the tumor–node–metastasis (TNM) staging of the disease [71]. These metabolic markers provide potent non-invasive diagnostic strategies; for instance, C6-DC alone yielded an AUC of 0.813, which further improved to 0.837 when combined with C4-OH. This suggests that panels featuring dicarboxylic and hydroxylated AC species can significantly enhance the precision of colorectal cancer screening and staging [71].

These reports indicate that colorectal cancer progression is accompanied by perturbations in short- and medium-chain ACs that mirror imbalances in mitochondrial metabolic flux, supporting AC-based panels for screening and staging.

#### 4.2.3. Pancreatic Cancer

AC profiles serve as critical differentiators between subtypes, with C2 concentrations distinguishing pancreatic neuroendocrine tumors (PNETs) from pancreatic ductal adenocarcinoma (PDAC) [74,75]. To address the persistent challenge of early detection, researchers have utilized (iso)butyrylcarnitine in pancreatic cyst fluid as a high-accuracy marker for identifying malignancy. This metabolite exhibits 28-fold elevations in malignant cysts, achieving a diagnostic accuracy of 89% [76]. Additionally, C2 has emerged as a high-potential early detector with an AUC of 0.93 [74].

Overall, subtype-associated AC perturbations can aid discrimination between malignant and non-malignant lesions, supporting the potential role of AC profiling in early detection and diagnostic refinement.

#### 4.2.4. Gastric Cancer

Gastric cancer is characterized by significant elevations in C6, C6-DC, and C16-OH, reflecting a leaky version of FAO where hydroxylated and dicarboxylic intermediates are released due to mitochondrial overload [77,78]. These biochemical shifts have been integrated into highly effective diagnostic models; a serum panel comprising C0, select ACs (C6, C6-DC, C16-OH), and arginine distinguished gastric cancer from gastritis with an AUC of 0.9977. Furthermore, a dried blood spot (DBS) panel including C4-OH, the C5/C3 ratio, and C10:2 achieved an AUC of 0.9318, demonstrating the clinical potential of AC-based screening [77,78].

In line with these findings, elevations in hydroxylated and dicarboxylic AC species are consistent with incomplete FAO under mitochondrial overload, and the reported models highlight AC-based approaches for sensitive detection and metabolic screening.

### 4.3. Respiratory and Aerodigestive Cancers: Signatures Driven by Environmental Stress and Metabolic Flux

#### 4.3.1. Lung Cancer

C16 stands out as a highly sensitive marker for lung cancer diagnosis [79]. The metabolic landscape is further refined by smoking status; for instance, nonsmokers show distinct risk associations with C12 and C4-OH, whereas smokers exhibit a depletion of C3 and C5:1, indicating that tobacco-induced stress significantly alters systemic AC homeostasis [80,81,82]. In metastatic non-small cell lung cancer (NSCLC), a marked depletion of medium-chain ACs suggests an exhaustive consumption of these substrates to fuel invasive behavior [83]. These flux-driven signatures have been successfully integrated into advanced diagnostic models. For instance, an XGBoost machine learning model incorporating amino acids and eight specific ACs, including C4-DC, C4-OH, C5, C5-DC, C12, C16, C22, and C26, attained an AUC of 0.81. This performance surpassed conventional clinical nomograms in accuracy, demonstrating the significant potential of AC-inclusive predictive technologies [84].

Lung cancer therefore shows AC signatures influenced by environmental exposure, smoking status, and metastatic progression, supporting AC-inclusive metabolic profiling as a component of risk prediction and model development.

#### 4.3.2. Nasopharyngeal Carcinoma (NPC)

NPC is characterized by intense metabolic demands, leading to a marked increase in serum and urinary concentrations of medium-chain ACs, specifically C8 and C10 [85]. These species serve as robust indicators of the metabolic reprogramming required for NPC progression. Clinically, a diagnostic panel comprising C8, C10, and creatinine demonstrated exceptional performance, yielding an AUC of 0.973. This highlights the potential of using medium-chain ACs as non-invasive tools for the precise detection of NPC [85].

NPC shows elevations in medium-chain AC species that align with heightened metabolic demand during progression, supporting AC profiling as a non-invasive diagnostic approach.

### 4.4. Specialized AC Signaling: Orchestrating Metastasis and Microenvironment Crosstalk

Beyond their roles in energy production, specific AC species function as active signaling entities that mediate tumor progression and systemic crosstalk.

#### 4.4.1. Biliary Tract Cancer and Gallbladder Cancer

Biliary tract cancer manifests distinct AC perturbations driven by metabolic reprogramming. Specifically, significant elevations in long-chain AC species, notably C16, have been demonstrated in the serum of patients. This systemic accrual, reflecting altered FAO flux, serves as a critical discriminatory biomarker against benign biliary strictures, thereby supporting the clinical utility of AC profiling in these malignancies [86].

In gallbladder cancer, a unique regulatory mechanism involves the LncBCL2L11-THOC5 axis, which drives the accumulation of long-chain ACs such as C18. Rather than being sequestered for β-oxidation, this accumulated C18 acts as a signaling ligand that triggers c-Jun N-terminal kinase (JNK) phosphorylation. This molecular event establishes a positive feedback loop that enhances tumor cell motility and promotes widespread metastasis, illustrating how ACs can directly dictate oncogenic cell behavior [87].

These observations point to a signaling-centric role of long-chain ACs in biliary tract and gallbladder cancers. Systemic elevation of C16 may aid discrimination from benign strictures, while intracellular C18 signaling has been linked to pro-metastatic behavior, extending the relevance of ACs beyond transport intermediates.

#### 4.4.2. Melanoma

Metabolic shifts are closely tied to disease progression and the systemic environment. Advanced cases exhibit a significant enrichment of C5-DC and C14:2, which are predictive of a poor prognosis [88]. A diagnostic model incorporating DL-carnitine and C8 identified advanced melanoma with an AUC of 0.822 and 100% sensitivity, offering a powerful method for clinical staging [89]. These AC fluctuations inversely correlate with the abundance of specific gut microbes, such as Faecalibacterium prausnitzii, suggesting that the systemic AC profile in melanoma may be influenced by the gut-metabolism axis [88].

Melanoma progression has been associated with stage-linked changes in specific AC species and with systemic metabolic interactions, including correlations with gut microbial composition. These patterns support the concept that AC profiling can capture both tumor-associated metabolism and host microenvironment crosstalk relevant to clinical stratification.

As summarized in Table 1, AC signatures across malignancies show recurring associations between chain-length patterns and clinical context, but the direction and magnitude of changes vary by cancer type, cohort, and sample matrix. Short-chain species are often reported in early-detection settings, whereas medium- and long-chain changes are frequently described in relation to progression, metabolic stress, and FAO engagement; however, these trends should be interpreted cautiously given cross-study variability [90,91,92]. AC profiles also differ by specimen type, with distinct signatures reported between tissue and biofluids, and conflicting findings for selected species likely reflect tumor heterogeneity as well as analytical and pre-analytical differences. These issues highlight the need for large-scale validation and standardized protocols before AC-based biomarkers can be considered for routine clinical use, and for clear separation between exploratory signals and clinically validated applications.

The inherent heterogeneity in pre-analytical sample handling, analytical platforms, data normalization strategies, and AC annotation pipelines constrains cross-study comparability and remains a formidable obstacle to the clinical translation of AC-based biomarkers.

## 5. Alteration in AC Metabolism and Therapeutic Targets

Metabolic reprogramming frequently confers resistance to conventional chemotherapeutics by modulating drug efflux pumps or fortifying survival signaling. Recent comprehensive reviews underscore that targeting these metabolic adaptations can re-sensitize resistant tumors to standard treatment regimens [94]. This section highlights key findings on how AC axis related metabolic proteins and pathways represent actionable targets for therapeutic intervention.

### 5.1. CPT System

As key gatekeepers of mitochondrial fatty acid entry, CPT inhibitors have demonstrated anticancer activity in preclinical models and early clinical settings. Etomoxir (ETO), a CPT1 inhibitor, combined with temozolomide (TMZ) reduced glioblastoma (GBM) stemness and invasiveness while prolonging survival in murine models [95,96]. Recently, teglicar (ST1326), a novel CPT1A inhibitor, demonstrated 92% cytotoxicity in canine mammary carcinoma cell lines without significant toxicity. This facilitates the translation of such findings from preclinical studies to veterinary oncology [97].

Furthermore, perhexiline enhanced cisplatin sensitivity in high-grade serous ovarian cancer by suppressing both CPT1A and CPT2 activities [98,99].

Inhibition of CPT1 disrupts FAO pathways, thereby retarding tumor growth and improving therapeutic responses. Especially, CPT1A blockade sensitizes NPC cells to radiotherapy [93,100]. Additionally, combining CPT1 inhibition with immune checkpoint blockade bolsters antitumor immunity within the TME [47].

CPT inhibition therefore perturbs mitochondrial fatty acid entry and AC-dependent flux, limiting FAO linked energy supply and stress adaptation, and can potentiate responses to chemotherapy, radiotherapy, and immunotherapy.

### 5.2. Uncoupling Protein 2 (UCP2)

UCP2, located in the mitochondrial inner membrane, plays a critical role in regulating the electron transport chain and maintaining redox balance. Numerous studies have reported its contribution to alleviating oxidative stress within cancer cells [101]. Significantly, the overexpression of UCP2 across various cancer types mediates the suppression of oxidative stress and facilitates metabolic reprogramming, thereby promoting tumor cell proliferation and resistance to chemotherapy [102,103].

Genipin acts as a specific UCP2 inhibitor and enhances the efficacy of trastuzumab treatment in human epidermal growth factor receptor 2 (HER2)-positive breast cancer cells (BT474). It achieves this by lowering the half maximal inhibitory concentration (IC50) approximately tenfold and synergistically increasing apoptosis [101]. Moreover, UCP2 inhibition suppresses proliferation and improves drug sensitivity in several other malignancies, including pancreatic, gallbladder, and NSCLC [104,105]. Studies also indicate that UCP2 functions as a transporter of C4 metabolites, such as malate and aspartate, from mitochondria to the cytosol. This process facilitates effective glutamine utilization by cancer cells, thereby contributing to metabolic adaptations such as the Warburg effect [106,107].

UCP2 can support cancer cell survival by modulating redox balance and metabolic adaptation under oxidative stress. Targeting UCP2 may destabilize mitochondrial homeostasis and improve therapeutic efficacy in combination settings.

### 5.3. OCTN2

OCTN2 is pivotal for the cellular uptake and metabolism of carnitine. It is markedly overexpressed in GBM tumor cells, where it closely associates with tumor growth and therapeutic resistance. Meldonium effectively suppresses OCTN2 activity to block carnitine transport, thereby disrupting FAO pathways within cancer cells. This anti-tumor mechanism was validated in GBM mouse models, where the daily administration of meldonium (250 mg/kg over 14 days) led to substantial tumor growth inhibition relative to controls [108].

Specifically, meldonium exerts its antitumor effects by simultaneously inhibiting the L-carnitine biosynthesis enzyme gamma-butyrobetaine hydroxylase 1 and the transporter OCTN2, which leads to the depletion of intracellular L-carnitine pools. This collapse of mitochondrial fatty acid metabolism induces energy starvation and oxidative stress, ultimately promoting cancer cell death [108].

Recent clinical case reports confirm these effects. The administration of meldonium to three patients with end-stage recurrent GBM who had failed standard therapies demonstrated excellent tolerability. In some cases, it also resulted in tumor growth suppression and survival extension, suggesting its potential as a metabolic targeted therapy [109].

OCTN2 mediated carnitine transport sustains FAO and can support tumor growth and therapeutic resistance. Pharmacological disruption of this transporter induces metabolic stress, highlighting OCTN2 as a viable target for metabolic intervention.

### 5.4. CACT

CACT, a transporter located in the mitochondrial inner membrane, facilitates the exchange of ACs and carnitine between the mitochondrial matrix and the cytosol, playing an essential role in fatty acid β-oxidation [11]. The inhibitor ST1326 targets both CPT1A and CACT, thereby blocking FAO and impairing mitochondrial fatty acid transport. This inhibition precipitates lipid accumulation and mitochondrial dysfunction in Burkitt lymphoma cells, leading to reduced cell viability. Importantly, ST1326 selectively targets c-Myc overexpressing lymphoma cells, suppressing their survival and impeding lymphoma development [110].

In hepatocellular carcinoma, CACT expression is downregulated due to the overexpression of miR-132-3p, a phenomenon associated with poor patient prognosis. The reduction in CACT leads to impaired FAO, which in turn promotes tumor cell growth and metastasis while adversely affecting cell cycle progression and apoptosis regulation [111].

Additionally, recent investigations have identified CACT as a pivotal promoter of pancreatic adenocarcinoma progression, particularly under lipid-rich dietary conditions. Pancreatic cancer cells rely heavily on CACT-mediated FAO to compensate for energy deficits. Inhibiting this transporter disrupts fatty acid entry into the mitochondria, triggers energy stress, and abolishes tumor promotion linked to high-fat diets [112].

CACT regulates mitochondrial AC exchange and FAO flux, supporting cancer cell plasticity under nutrient stress. Targeting CACT can expose selective metabolic vulnerabilities and reinforces the therapeutic relevance of the FAO-AC axis.

**Figure 3:**
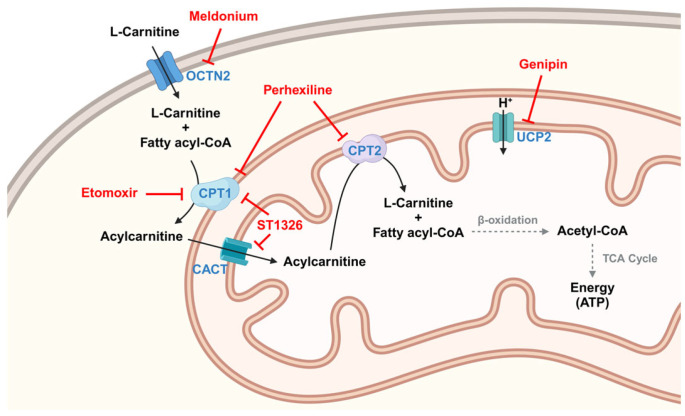
Schematic diagram of the production and β-oxidation process of AC and inhibitors of related molecules. Proteins are indicated in blue letters, metabolites are written in black letters, and inhibitors are shown in red letters. [Created in BioRender. Hwa Pyoung, L. (2026) https://BioRender.com/nwnwh9a].

As summarized in Table 2 and Figure 3, AC targeting strategies involving CPTs, UCP2, OCTN2, and CACT represent metabolic checkpoints that control cancer cell plasticity, chemoresistance, and survival under nutrient stress. Inhibitors within the FAO-AC axis possess the potential to enhance the efficacy of radiotherapy, chemotherapy (such as TMZ and cisplatin), and immunotherapy. The selective vulnerabilities observed in c-Myc overexpressing lymphomas and lipid-dependent pancreatic cancers highlight the potential of AC profiling as a companion diagnostic tool. However, successful clinical translation necessitates the development of selective, non-toxic inhibitors and the rigorous validation of synergistic combination therapies.

## 6. Challenges and Future Directions

### 6.1. From Static Pool Sizes to Functional Flux

A pivotal challenge remains that AC measurements primarily capture pool sizes rather than FAO flux. Mechanistic inference therefore requires alignment with functional assays that define mitochondrial throughput and respiratory constraints, for example, stable isotope tracing and mitochondrial respiration measurements [27]. However, interpreting these changes is complicated by the profound adaptability of cancer metabolism, which can reallocate substrate use across pathways under stress. This necessitates sophisticated analytical approaches to address these challenges effectively [6,113].

### 6.2. Addressing Systemic Confounders and Standardization

The diagnostic accuracy of AC profiling is markedly impacted by differences in sample collection sites, such as plasma, serum, or tissue. Establishing a clear distinction between non-invasive and invasive sampling methods is imperative, as such differentiation serves as a prerequisite for providing appropriate context in metabolomic data interpretation [114].

Beyond pre-analytical variability, a primary challenge stems from the fact that circulating AC levels reflect the aggregate of whole-body metabolic flux. As a result, AC metabolic activity varies significantly among individuals based on environmental and host factors. It is essential to consider patient-specific characteristics including body weight, fat distribution, and overall health status, as these variables directly influence systemic metabolic regulation [59]. For instance, as skeletal muscle serves as the principal reservoir of carnitine, baseline AC pools are prone to perturbations from physical activity or cancer-associated sarcopenia, which potentially obscure tumor-derived metabolic signals [9,115]. In addition, hepatic dysfunction, which is prevalent in advanced malignancies, may introduce confounding variables through impaired systemic lipid processing [69]. Moreover, gut microbiota-mediated metabolism of dietary carnitine into trimethylamine-N-oxide (TMAO) modulates systemic bioavailability, thereby introducing non-tumorigenic noise [116].

To mitigate these systemic confounders, the adoption of rigorous sampling protocols, including standardized fasting durations, time-of-day harmonization, and the exclusion of subjects with severe metabolic comorbidities, is imperative [117,118]. Moreover, transitioning the analytical paradigm from absolute quantification toward ratio-based interpretation (e.g., specific AC-to-free carnitine ratios) or longitudinal monitoring to establish personalized baselines can effectively enhance the specificity of these biomarkers for tumor detection.

### 6.3. Defining Therapeutic Windows and Mitigating Systemic Toxicity

Although targeting the AC-FAO axis presents a compelling therapeutic strategy, its clinical advancement is often constrained by the fundamental reliance of normal tissue homeostasis on FAO. FAO functions as the primary bioenergetic engine for tissues with high metabolic demand, most notably the myocardium and skeletal muscle [9,10]. Hence, systemic inhibition of CPT or CACT entails a substantial risk of on-target toxicity, potentially triggering cardiac lipotoxicity or myopathy capable of phenocopying inherited carnitine cycle defects [119].

Metabolic plasticity also extends to immune effector cells, particularly memory T cells, which depend heavily on FAO for their survival and functional differentiation [120]. This engenders a therapeutic paradox: while CPT1A inhibition has been demonstrated to invigorate anti-tumor immunity by alleviating the tumor-mediated immunosuppressive microenvironment [47], indiscriminate blockade carries the theoretical risk of compromising the metabolic fitness of memory T cells. Currently, the therapeutic window (defined as the dosage range that effectively restricts neoplastic FAO dependence while preserving oxidative capacity in healthy tissues) remains insufficiently characterized. Future investigations must, therefore, prioritize the development of tumor-selective delivery systems or allosteric modulators that exploit subtle kinetic distinctions between neoplastic and physiological FAO isoforms and incorporate translational safety frameworks, including tissue-specific pharmacodynamic markers and cardiac monitoring strategies [121]. Additionally, addressing potential off-target effects of current FAO inhibitors remains crucial to mitigating unintended metabolic interference.

### 6.4. Spatial Metabolomics and Intratumoral Heterogeneity

Traditional bulk metabolomic analyses average metabolite levels across entire tumor specimens, thereby obscuring intratumoral spatial heterogeneity that arises from microenvironmental gradients such as hypoxia, immune infiltration, and proliferative zoning. Spatial metabolomics, specifically mass spectrometry imaging (MSI), facilitates the in situ visualization of metabolite distributions across tissue sections, revealing metabolic differences between distinct tumor subregions that bulk methods cannot resolve [122,123]. MSI integrates metabolite detection with tissue histology, effectively preserving anatomical context and enabling the comparison of metabolic states across heterogeneous regions.

Spatially resolved profiling has elucidated metabolic reprogramming in cancer. For instance, matrix-assisted laser desorption/ionization (MALDI) MSI of human breast cancer tissues and xenograft models visualized spatial patterns of carnitine and AC species, including significant alterations in L-carnitine and several short-chain and long-chain ACs within tumor versus adjacent normal areas [124]. This investigation also identified differential expression of key enzymes such as CPT1A, CPT2, and carnitine O-acetyltransferase (CRAT) within specific regions, correlating spatial metabolite distribution with localized enzyme expression and implying region-dependent FAO activity [124].

In addition to single metabolite mapping, spatial metabolomics has been applied at large scale. In gastric cancer, high mass resolution MSI identified distinct metabolic subtypes within tumor tissue based on spatial clustering of metabolites, linking localized metabolic signatures to molecular features and clinical outcomes, including differential responses to trastuzumab therapy across subtypes [125]. Such spatial subtypes would remain undetectable in bulk analyses and underscore the importance of spatial context in interpreting metabolic phenotype and therapeutic response.

These studies demonstrate that AC distributions and metabolic profiles more broadly vary across tumor microregions, encompassing differences potentially associated with FAO activity and microenvironmental stresses. Spatial metabolomics provides an essential framework for elucidating such heterogeneity, enabling deeper mechanistic insight into localized metabolic adaptations during cancer progression and therapy response that bulk metabolomics cannot resolve.

### 6.5. Dynamic Tracking and Spatial Multi-Omics

Tumor cells often circumvent therapeutic stress through metabolic pathway rerouting, a process frequently mediated by AC metabolic regulators. Therefore, long-term and multidimensional tracking of these regulators is imperative [113,126]. Integrative multi-omics approaches, combining spatial metabolomics with transcriptomics, are expected to delineate AC-centered tumor-stroma interactions in unprecedented detail. Recent mass spectrometry imaging studies have provided significant insights. For instance, spatially resolved metabolomics has visualized the reprogramming of carnitine metabolism in breast cancer [124], while spatial single-cell isotope tracing has revealed the inherent heterogeneity of de novo fatty acid synthesis across cell populations within the TME [127]. These advancements demonstrate that AC gradients at tumor invasion fronts can serve as powerful prognostic spatial signatures in specific contexts [128].

### 6.6. High-Dimensional Data Analytics and Patient Stratification

The ongoing integration of big data analytics and artificial intelligence is accelerating the development of AC-based precision diagnostics [129,130,131,132,133]. Beyond technical advancements, the practical application of AC profiling in precision oncology provides a robust framework for enhancing the precision of personalized cancer management. ACs could serve as pivotal tools for companion diagnostics, enabling the selection of patients most likely to benefit from specific FAO-targeted therapies, such as CPT1/2 inhibitors. By monitoring real-time changes in systemic AC levels, clinicians can achieve a dynamic assessment of therapeutic efficacy and the early detection of drug resistance, ensuring that interventions are tailored to the unique bioenergetic demands of each patient’s tumor.

### 6.7. Roadmap for Clinical Translation

Despite these technological strides, several critical challenges remain. Standardizing mass spectrometry imaging protocols for routine clinical settings and resolving the sub-cellular localization of specific AC pools are essential future steps. Integrating spatial data with functional metabolic flux analysis will also be indispensable to fully decode the complex role of ACs within the tumor ecosystem. However, a primary bottleneck in clinical translation is the disproportionate reliance on in vitro systems and murine models, which frequently fail to faithfully recapitulate the complex metabolic heterogeneity, immune interactions, and pharmacokinetics observed in humans. To address these limitations, future investigations should prioritize the integration of patient-derived organoids (PDO) and humanized models to more accurately reproduce the human TME [134]. Looking ahead, future research must prioritize large-scale collaborations involving diverse patient cohorts to ensure the broad applicability of these findings. This requires the development of standardized protocols for sample handling and data analysis, alongside transparent data-sharing frameworks. Concurrently, rigorous clinical validation and regulatory approval processes are necessary to successfully translate AC-based biomarkers from laboratory research into daily clinical practice [113,126].

### 6.8. Translational Limitation of AC-Based Biomarkers and Therapies

Although acylcarnitine (AC) profiling shows promise as a metabolic biomarker in cancer research, the current evidence is largely preclinical or based on modest clinical cohorts, with limited large-scale prospective validation. Targeted metabolomics studies have reported that serum AC profiles can discriminate patients with multiple solid tumors from healthy controls, but these reports are constrained by small sample sizes and retrospective designs, supporting exploratory diagnostic potential rather than validated clinical utility [35]. Similarly, case–control metabolomic profiling has identified differential serum ACs in gastric and lung cancer cohorts; however, these findings are mainly from single-center studies and lack multicenter or longitudinal validation [77,135].

Mechanistic reviews highlight the importance of AC metabolism, particularly the roles of β-oxidation and mitochondrial fatty acid transport in shaping circulating AC dynamics, yet AC alterations observed in human cancer populations remain largely descriptive, without prospectively established diagnostic performance or outcome prediction thresholds [92,136]. In addition, circulating AC levels are influenced by systemic factors beyond tumor biology, including diet, fasting status, comorbid metabolic disease, and renal handling, which can confound interpretation in heterogeneous clinical populations [136].

Clinical translation of AC-targeting therapies also remains early-stage. CPT1 inhibition (e.g., etomoxir) and broader FAO blockade show anti-tumor effects in murine models, but these approaches have not achieved widespread clinical adoption because of systemic toxicity concerns and the absence of randomized phase II/III trials in cancer populations [137,138]. Variable selectivity and off-target liabilities of available FAO inhibitors further complicate clinical development [139].

In contrast to the large-scale, multicenter clinical validation required for regulatory endorsement, most oncology studies of AC biomarkers rely on constrained cohorts with limited external validation. Addressing this gap will require prospective, standardized cohort studies with harmonized preanalytical handling and rigorous statistical modeling to evaluate diagnostic and prognostic performance across diverse populations. Until such evidence is established, AC-based diagnostics and therapeutic strategies should be regarded as emerging translational concepts rather than established clinical tools.

## 7. Conclusions

ACs occupy a central position in cancer metabolism by influencing energy production, immune regulation, and therapeutic resistance. Alterations in AC levels across various cancer types correlate closely with tumor initiation, progression, prognosis, and treatment response. These metabolic shifts possess significant potential as non-invasive diagnostic biomarkers that reflect the underlying metabolic state of the malignancy. Recent advances in high-performance analytical techniques and artificial intelligence algorithms have markedly improved diagnostic accuracy and subtype classification. Additionally, proteins and pathways related to AC metabolism are emerging as promising therapeutic targets for overcoming resistance and suppressing tumor growth.

In essence, the translation of ACs from laboratory markers to bedside tools will redefine the clinical landscape of oncology. By integrating multidimensional metabolomic data with patient-specific clinical profiles, AC-based platforms will facilitate a more nuanced understanding of tumor plasticity. This integration not only enhances diagnostic precision but also empowers the development of innovative, metabolism-centered treatment regimens in appropriately validated settings.

Research on ACs offers critical insights into the complexity and heterogeneity of cancer metabolism, serving as an essential component for the implementation of personalized precision medicine. However, it is imperative to acknowledge that FAO and AC dependencies exhibit significant heterogeneity across diverse cancer types, stages, and microenvironments. Given that insights gleaned from specific tumor models may lack broad generalizability, delineating this metabolic plasticity is paramount to circumventing oversimplification and facilitating the efficacious integration of personalized precision medicine. Future progress that integrates multidimensional metabolomics with clinical data, alongside advances in spatial metabolomics, will further accelerate the development of AC-based diagnostics and therapies. Consequently, ACs are expected to establish a new paradigm in cancer management, offering high clinical applicability that spans early diagnosis, tailored treatment, and prognostic evaluation provided that analytical and clinical validation requirements are met. Therefore, continued in-depth investigation into AC biology will contribute to improving cancer patient survival and quality of life, ultimately establishing AC-related tools as indispensable components in clinical oncology practice.

## Figures and Tables

**Table 1:** Cancer-specific AC alterations and their clinical relevance.

Metabolic Category	Cancer Type	Sample Type	Increased ACs	Decreased ACs	Clinical Application	Ref.
Glandular and Reproductive	Ovarian cancer	Non-invasive (Plasma)	C2, C3	C8, C10:1	Diagnosis, Disease risk, Advanced clinical stage, Staging, Tumor progression	[55,56]
		Invasive (Tissue)	C2, C4	-	Preliminary cancer diagnosis and metastatic disease progression	[57]
	Breast cancer	Non-invasive (Plasma/ Serum/DBS)	C2, C4, C5, C5-M-DC	C3-DC, C10:1, C10:2	Risk assessment, Early diagnosis, Subtypes (ER-positive risk), Screening	[59,60,61]
	Prostate cancer	Non-invasive (Plasma)	C4, medium-chain ACs (C6-C12)	Long-chain ACs (C14-C16)	Pathological staging, Prognostic evaluation, Disease advancement, Metastasis progression	[63,65]
		Invasive (Tissue)	C16	-	Prognostic evaluation	[64]
Digestive and Hepatobiliary	Hepato cellular carcinoma	Non-invasive (Plasma/ Serum)	C2 *, C4-DC, C6 *, C14:1 *, C18:1, C18:2	C2 *, C5, C6 *, C8, C8:1, C10, C10:1, C12, C14:1 *, C18	Early diagnosis, Progression tracking, Etiology-specific diagnosis	[66,67,68,69,70]
	Colorectal cancer	Non-invasive (Serum)	C2, C14, C14:1, C14:2, C16:1, C6-DC, C8-DC, C4-OH, C14-OH, C16-OH, C18-OH, C18:1-OH	C11	Diagnosis, Staging assessment, Progression stages, Distinguishing adenoma from carcinoma	[71,72]
		Invasive (Tissue)	C4, C14, C16, C18, C18:1	-	Diagnosis	[73]
	Pancreatic cancer	Non-invasive (Serum)	-	C2, C14:1, C14:2	Progression, Subtyping (PDAC vs. PNET), Prognostic insights, Metastasis correlation	[74,75]
		Invasive (Cyst)	(Iso)butyrylcarnitine	-	Early detection	[76]
	Gastric cancer	Non-invasive (Serum, DBS)	C0, C3-DC, C4-OH, C6, C6-DC, C16-OH, C18:1	C10:2	Diagnosis, Distinguishing cancer from gastritis	[77,78]
Respiratory and Aerodigestive	Lung cancer	Non-invasive (Plasma/ Serum)	C3 *, C4, C5, C12, C14, C16	C3 *, C5:1, C4-OH, C26	Diagnosis, Risk assessment, Smoking status-specific risk, Early-stage disease indicators	[79,82]
		Invasive (Tissue)	C20, C22	C2-C16	Subtype distinction (Adeno/Squamous), Progression (Metastatic vs. Primary)	[81]
	Naso pharyngeal carcinoma	Non-invasive (Serum, Urine)	C8, C10	-	Diagnosis, Monitoring	[85,93]
Specialized AC Signaling	Melanoma	Non-invasive (Serum)	C7-DC, C14:2, C18:1	C0, C3, C4, C5-M-DC, C5-OH, C5:1, C5:1-DC	Diagnosis, Predicting cancer outcomes, Risk stratification, Prognostic assessment, Identifying advanced cases	[88,89]

Asterisks (*) in the “Increased ACs” or “Decreased ACs” columns indicate that conflicting reports (some studies showing an increase, others showing a decrease) exist in the literature regarding the concentration of that specific AC for the corresponding cancer type.

**Table 2:** Therapeutic targeting of AC metabolism in cancer.

Target	Anticancer Agent	Mechanism (Action Summary)	Cancer Type	Key Findings (Clinical/Biological Benefit)	Ref.
CPT1/2	ETO + TMZ	Inhibits CPT1/2; Blocks FAO; Suppresses TCA/ATP	GBM	Reduced viability/ATP; Reduced stemness/invasiveness; Prolonged survival	[95,96]
CPT1A + CPT2	Perhexiline + Cisplatin	Inhibits CPT1A/CPT2; Blocks FAO	High-grade serous ovarian cancer	Restored cisplatin sensitivity; Increased chemo efficacy	[98,99]
CPT1	ETO	Blocks CPT1; Disrupts FAO	NPC	Radiotherapy sensitization	[93,100]
UCP2	Genipin + Trastuzumab	Inhibits UCP2; Enhances HER2 efficacy; Suppresses oxidative stress	HER2-positive breast cancer (BT474 cell line)	Increased apoptosis; IC50 lowered tenfold	[101]
UCP2	Genipin	Inhibits UCP2	Pancreatic, Gallbladder, NSCLC	Suppressed proliferation; Improved drug sensitivity	[104,105]
OCTN2	Meldonium	Inhibits OCTN2/BBOX1; Depletes Carnitine; Impairs FAO	GBM	Substantial tumor growth inhibition; Prolonged survival	[109]
CACT	ST1326	Inhibits CPT1A/CACT; Blocks FAO; Impairs AC transport	Burkitt lymphoma (c-Myc-overexpressing)	Selective survival suppression; Reduced viability via lipid accumulation	[110]

## Data Availability

No new data were created or analyzed in this study. Data sharing is not applicable to this article.

## Abbreviations

The following abbreviations are used in this manuscript:

AC	Acylcarnitine
ACC	Acetyl-CoA Carboxylase
AFP	Alpha-Fetoprotein
AMPK	AMP-activated protein kinase
ATP	Adenosine Triphosphate
AUC	Area Under the Curve
BBOX1	Gamma-Butyrobetaine Hydroxylase 1
C0	Free Carnitine
C2	Acetylcarnitine
C3	Propionylcarnitine
C3-DC	Malonylcarnitine
C4	Butyrylcarnitine
C4-DC	Methylmalonylcarnitine; Succinylcarnitine
C4-OH	Hydroxybutyrylcarnitine
C5	Isovalerylcarnitine; 2-Methylglutarylcarnitine
C5-DC	Glutarylcarnitine
C5-M-DC	3-Methylglutarylcarnitine
C5-OH	Hydroxyisovalerylcarnitine
C5:1	Isopentenoylcarnitine; Tiglylcarnitine
C5:1-DC	Glutaconylcarnitine
C6	Hexanoylcarnitine
C6-DC	Adipoylcarnitine
C7-DC	Pimelylcarnitine
C8	Octanoylcarnitine
C8-DC	Suberylcarnitine
C8:1	Octenoylcarnitine
C10	Decanoylcarnitine
C10:1	Cis-4-Decenoylcarnitine; Decenoylcarnitine
C10:2	Decadienoylcarnitine
C11	O-(4,8-Dimethylnonyl)carnitine
C12	Dodecanoylcarnitine
C14	Myristoylcarnitine; Tetradecanoylcarnitine
C14-OH	Hydroxytetradecanoylcarnitine
C14:1	Tetradecenoylcarnitine
C14:2	Tetradecadienoylcarnitine
C16	Palmitoylcarnitine
C16-OH	Hydroxyhexadecanoylcarnitine; Hydroxypalmitoylcarnitine
C16:1	Palmitoleoylcarnitine
C18	Stearoylcarnitine
C18-OH	Hydroxyoctadecanoylcarnitine
C18:1	Octadecenoylcarnitine; Oleoylcarnitine
C18:1-OH	Hydroxyoctadecenoylcarnitine
C18:2	Linoleoylcarnitine; Octadecadienoylcarnitine
C20	Eicosylcarnitine
C22	Behenoylcarnitine
C26	Hexacosanoylcarnitine
CACT	Carnitine–Acylcarnitine Translocase (SLC25A20)
c-Myc	MYC Proto-Oncogene
CoA	Coenzyme A
CPT	Carnitine Palmitoyltransferase
CPT1	Carnitine Palmitoyltransferase 1
CPT1A	Carnitine Palmitoyltransferase 1A
CPT2	Carnitine Palmitoyltransferase 2
CRAT	Carnitine O-Acetyltransferase
DBS	Dried Blood Spot
ER+	Estrogen Receptor-Positive
ETO	Etomoxir
FADH2	Flavin Adenine Dinucleotide
FAO	Fatty Acid Oxidation
GBM	Glioblastoma
HER2	Human Epidermal Growth Factor Receptor 2
IC50	Half Maximal Inhibitory Concentration
JNK	c-Jun N-terminal Kinase
MALDI	Matrix-Assisted Laser Desorption/Ionization
MSI	Mass Spectrometry Imaging
NADH	Nicotinamide Adenine Dinucleotide
NADPH	Nicotinamide Adenine Dinucleotide Phosphate
NF-κB	Nuclear Factor Kappa B
NPC	Nasopharyngeal Carcinoma
NRF2	Nuclear Factor Erythroid 2–Related Factor 2
NSCLC	Non-Small Cell Lung Cancer
OCTN2	Organic Cation/Carnitine Transporter 2 (SLC22A5)
PDAC	Pancreatic Ductal Adenocarcinoma
PDO	Patient-Derived Organoid
PNET	Pancreatic Neuroendocrine Tumor
ROS	Reactive Oxygen Species
ST1326	Teglicar
STAT3	Signal Transducer and Activator of Transcription 3
TCA	Tricarboxylic Acid Cycle
TMAO	Trimethylamine-N-oxide
TME	Tumor Microenvironment
TMZ	Temozolomide
TNM	Tumor–Node–Metastasis
UCP2	Uncoupling Protein 2
